# Application of a Drug-Induced Apoptosis Assay to Identify Treatment Strategies in Recurrent or Metastatic Breast Cancer

**DOI:** 10.1371/journal.pone.0122609

**Published:** 2015-05-29

**Authors:** Linda Bosserman, Karl Rogers, Carl Willis, Dirk Davidson, Pat Whitworth, Misagh Karimi, Gargi Upadhyaya, James Rutledge, Allan Hallquist, Mathieu Perree, Cary A. Presant

**Affiliations:** 1 Wilshire Oncology Medical Group-US Oncology, La Verne, CA, United States of America; 2 Nashville Oncology Associates, Nashville, TN, United States of America; 3 Tennessee Plateau Oncology, Crossville, TN, United States of America; 4 Nashville Breast Center, Nashville, TN, United States of America; 5 Data Vision, Dayton OH, United States of America; 6 DiaTech Oncology, Montreal, Canada and Nashville, TN, United States of America; Stavanger University Hospital, NORWAY

## Abstract

**Background:**

A drug-induced apoptosis assay has been developed to determine which chemotherapy drugs or regimens can produce higher cell killing in vitro. This study was done to determine if this assay could be performed in patients with recurrent or metastatic breast cancer patients, to characterize the patterns of drug-induced apoptosis, and to evaluate the clinical utility of the assay. A secondary goal was to correlate assay use with clinical outcomes.

**Methods:**

In a prospective, non-blinded, multi institutional controlled trial, 30 evaluable patients with recurrent or metastatic breast cancer who were treated with chemotherapy had tumor samples submitted for the MiCK drug-induced apoptosis assay. After receiving results within 72 hours after biopsy, physicians could use the test to determine therapy (users), or elect to not use the test (non-users).

**Results:**

The assay was able to characterize drug-induced apoptosis in tumor specimens from breast cancer patients and identified which drugs or combinations gave highest levels of apoptosis. Patterns of drug activity were also analyzed in triple negative breast cancer. Different drugs from a single class of agents often produced significantly different amounts of apoptosis. Physician frequently (73%) used the assay to help select chemotherapy treatments in patients, Patients whose physicians were users had a higher response (CR+PR) rate compared to non-users (38.1% vs 0%, p = 0.04) and a higher disease control (CR+PR+Stable) rate (81% vs 25%, p<0.01). Time to relapse was longer in users 7.4 mo compared to non-users 2.2 mo (p<0.01).

**Conclusions:**

The MiCK assay can be performed in breast cancer specimens, and results are often used by physicians in breast cancer patients with recurrent or metastatic disease. These results from a good laboratory phase II study can be the basis for a future larger prospective multicenter study to more definitively establish the value of the assay.

**Trial Registration:**

Clinicaltrials.gov NCT00901264

## Introduction

The science of apoptosis has been widely studied and has been applied to the development of drugs targeting apoptosis mechanisms. Translation of this science to development of a predictive test for response to chemotherapy, in order to improve outcomes of chemotherapy, has been a goal of translational science and personalized medicine. Prior chemosensitivity/chemoresistance assays which have sought to predict clinical outcomes but which have not been direct measurements of drug induced apoptosis have performed poorly [1].

Recently, in blinded validity trials, a drug-induced apoptosis assay (called the Microculture Kinetic MiCK Assay) was found to correlate with patient outcomes in acute myelocytic leukemia [2], and ovarian cancer [3]. When the assay was initially studied in a broad utility trial in a heterogeneous patient population with various cancers, physicians who received results of the MiCK assay prior to deciding on chemotherapy were found likely to use the assay (64% usage) [4], and when physicians used the MiCK assay, there was an increase in complete or partial response rates, time-to-progression, and overall survival [5]. However, since the tumor diagnoses were heterogeneous, conclusions about the applicability of the drug induced apoptosis assay were tentative and awaited confirmatory studies in more homogeneous populations.

We therefore performed this follow up study of the drug induced apoptosis MiCK assay in patients with recurrent or metastatic breast cancer. This was a prospective multi-institutional, non-blinded study. The trial was listed on the CancerTrials.gov website (NCT00901264). The primary goal was to determine if the assay could be successfully performed with breast cancer cells from patient biopsies, and if physicians would use the assay results to direct therapy. The secondary goal was to correlate use of the assay with therapy choices and patient outcomes.

## Methods

The protocol for this trial and supporting CONSORT checklist are available as supporting information; see S1 CONSORT Checklist and S1 Protocol.

### The Drug Induced Apoptosis (MiCK) Assay Process

Tumor Cell Preparation: sterile tumor specimens (surgical biopsy, 5 core needle biopsies, or malignant effusion) are sent via overnight delivery to the DiaTech Oncology laboratory, Montreal, Quebec. Neoplastic cells are purified from the solid tumors, effusions or marrow by a series of steps (proprietary process, DiaTech Oncology)

After purification, each tumor cell preparation is analyzed by a pathologist using cytospin preparations and immunocytochemical stains. To be evaluable, each specimen must achieve at least 90% pure tumor cell content and 90% viability by trypan blue exclusion.

Human JURL-MK2 chronic leukemia in blast crisis cell line (DSMZ, Germany) is used as a positive control for MiCK assays performed with patient tumor cells. RPMI-1640 medium without phenol red is used, supplemented with 10% fetal bovine serum, 100 units/mL of penicillin, and 100 micrograms/mL of streptomycin. Cell counts and viability are evaluated by trypan blue dye exclusion.

MiCK assay for apoptosis: The MiCK assay procedure was adapted from the method described previously [6, 7]. After overnight incubation, chemotherapy drugs are added to 384-well plates in 2.5 (384 well) microliter aliquots. The number of drugs or drug combinations and the number of concentrations tested depend on the number of viable malignant cells that are isolated from the tumor specimen. The drug concentrations, determined by molarity, are those indicated by the manufacturer as the desired blood level concentration plus or minus one serial dilution if enough cells are available. Following drug addition, the plate is incubated for 30 min at 37° C in a 5% carbon dioxide humidified atmosphere incubator. Each well is then overlayed with sterile mineral oil, and the plate is placed into the incubator chamber of a microplate spectrophotometric reader (BioTek instruments). The apparent optical density at 600 nanometers is read and recorded every 5 minutes over a period of 48 hours. Apparent optical density increases, which correlate with apoptosis, are converted to kinetic units (KU) of apoptosis by a proprietary software ProApo with a formula described previously [8–11].

A typical apoptotic curve generated by the MiCK assay has an initial steep component followed by a plateau and/or decreasing portion of the curve when measuring OD vs time. The initial steep rise in the curve, where apoptosis is measured, is associated with the increased optical density. This is primarily associated with the blebbing of the cell membrane and, in part, to cytoplasmic and nuclear condensation. The resulting decline and/or plateau of the OD curve indicate various stages of cell disintegration. During apoptosis, there may be intercellular bridges apparent on phase contrast light microscopies which also contribute to the apparent optical density.

In the assay, active apoptosis is indicated as > 1.0 KU. A drug producing < = 1 KU is defined as inactive, and that the tumor cells are resistant to that drug (based on previous laboratory correlations of KU with other markers of drug-induced cytotoxicity such as growth in culture and thymidine uptake). The drugs or combinations which produce the best apoptosis in the assay are those with the highest KU value +/- 0.57 KU which is the standard deviation of the test (derived from repetitive assays in single tumor cell lines). Uses of any of the best therapies have correlated with statistically improved survival, time to relapse, and response rates in a prior trial in acute leukemia patients [2]. Results are sent to the submitting physician within 72 hours of specimen submission. Data on the coefficient of variation, accuracy, linearity, limits of detection, sensitivity and specificity are available as supplemental information. Further details of the methods of the assay have been published [6–11].

### Treatment of Patients

This study was a prospective multi-institutional non- blinded utility trial. MiCK assay results obtained before any therapy was initiated were always transmitted to physicians. Although the master protocol allowed any patient with cancer to be studied, it did not specify use of the assay in patients for adjuvant or palliative purposes. This communication studied only the subset of patients with recurrent or metastatic breast cancer. Physicians treated patients with the physicians’ own choice of drugs as they deemed clinically indicated and were free to use or not use any of the data from the MiCK assay. In this utility trial, tumor responses were measured by Response Evaluation Criteria in Solid Tumors or by reduction in sum of bi-dimensional tumor measurements at the discretion of the physician. Patients were evaluated for time to recurrence (defined as time from assay to progression by RECIST or measurement, or death from tumor) after assay and survival after assay.

Although there were no rules or directions regarding how to use the MiCK assay results, the pathologist suggested the most active drugs or combinations in the assay report. The study evaluated whether the oncologist used the results of the assay, whether other data were also used (e.g., estrogen receptor analysis or human epidermal growth receptor 2 [HER2] test results, or with the addition of other drugs which may or may not also have been on the MiCK assay results at various levels), or the assay results were not used. Because instructions or rules regarding using the assay were not given, it was felt that this was a more valid test of how the assay would be used in actual practice, where oncologist have complete discretion in treatment planning.

### Statistical Evaluation

The primary goal of the utility study was to identify how frequently physicians used the MiCK assay results to help determine patient treatment. The secondary goal of the utility study was to correlate use of the MiCK assay with response rate, relapse-free interval, and overall survival. Physicians completed questionnaires in which they described what the intended treatment was before the assay data were returned, what treatment was used after the assay was reported, and whether the assay was used in formulating the final treatment given to the patient. Data were imported into SAS software (SAS Institute, Cary, NC) for analysis. If a sample was tested with multiple doses of the same drug, then the concentration with the highest KU value was assigned to the drug. Nonparametric Kaplan-Meier product limit methods were used for survival analysis and the analysis of relapse-free interval. [12] In this analysis, the log-rank test was used to compare survival curves [13]. The Wilcoxon signed rank test was used for comparing differences between drugs in paired samples [14]. Response rates were compared using contingency tables and Pearson’s Chi square test [15].

### Investigational Review Board Approval

Investigators performed this trial after institutional review board (IRB) approval was obtained from and monitored by the Western IRB in Seattle, Washington. Each patient had given voluntary informed consent in writing before submission of tumor specimens for MiCK analysis.

## Results

### Patient Characteristics

**Fig 1:**
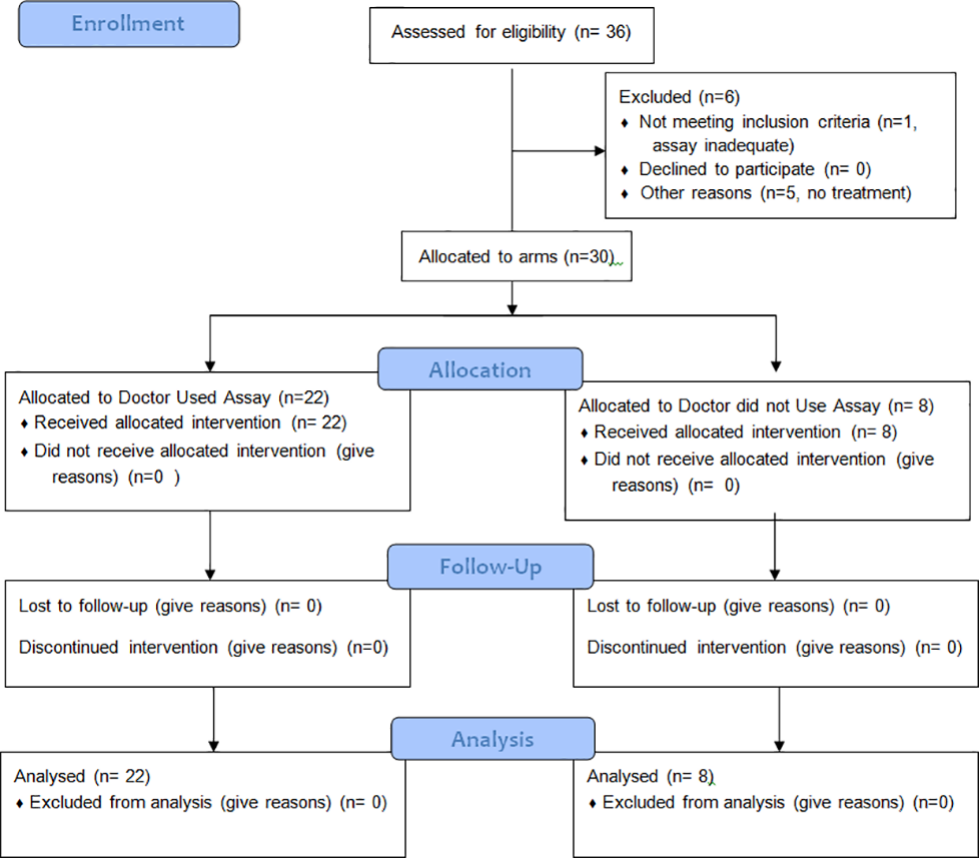
CONSORT Flow Diagram.

As indicated in Fig 1, the CONSORT diagram, 36 patients were entered, of which 30 were evaluable. The patient characteristics are listed in Table 1. Patients were scheduled to receive a median of third line chemotherapy after the assay, with a range of 1 to 8 lines of therapy. Patients generally had a good ECOG performance status. The assay was able to test a median of 12 drugs or combinations with a range of 3 to 31. More drugs were tested when effusions or biopsies were obtained, and fewer drugs were tested when the patients had only core biopsies submitted. The distribution of age, estrogen receptor, progesterone receptor, Her2 over-expression, number of lines of prior therapy, and centers treating the patients were not statistically different between patients in whom the MiCK assay was used to plan therapy versus patients in whom the assay was not used. There was a non-significant trend to later line of therapy to be used next in patients in whom the physician did not use the MiCK assay (4^th^ line median) versus earlier line of therapy to be used next in patients in whom the physician did use the MiCK assay (2^nd^ line median), p = 0.07. There was also a trend towards greater prior use of taxanes before the assay was performed in patients in whom the MiCK assay was not used (p = 0.07), and there was greater prior use of 5FU or capecitabine in those patients (p = 0.03).

**Table 1:** Patient Characteristics.

	ALL PATIENTS	USED MiCK	DID NOT USE MiCK	P-value MiCK usedvs Not Used
Number of patients	30	22	8	
Age—mean	59.4	60.2	57.1	0.42
Age—median	57	58.5	53.5	
Age—standard deviation (sd)	9	8.1	11.5	0.2
Age-range	42–81	42–74	45–81	
Lines of therapy—mean	2.6	2.1	4	0.07
Lines of therapy—median	2	2	4	
Lines of therapy—sd	1.8	1.2	2.4	0.01
Lines of therapy—range	1–8	1–5	1–8	
Ecog performance by proportion—ecog 0	12(40%)	11(50%)	1(12%)	0.16
Ecog performance by proportion—ecog 1	11(37%)	6(27%)	5(62%)	
Ecog performance by proportion—ecog 2	5(17%)	3(14%)	2(25%)	
Ecog performance by proportion—ecog 3	2(7%)	2(9%)	0(0%)	
Number of drugs tested—mean	14.3	15.3	11.2	0.43
Number of drugs tested—median	12	12	6	
Number of drugs tested—sd	9.9	10	10	0.87
Number of drugs tested—range	3–31	3–31	4–28	
Number of patients her2 positive	4(14%)	2(9%)	2(25%)	0.28
Prior therapy—hormonal therapy used	7(23%)	6(27%)	1(12.5%)	0.64
Prior chemotherapy – anthracyclines	17(57%)	12(55%)	5(63%)	1
Prior chemotherapy—platinums	8(27%)	6(27%)	2(25%)	1
Prior chemotherapy—taxanes	22(73%)	14(64%)	8(100%)	0.07
Prior chemotherapy—alkylating agents	17(57%)	13(59%)	4(50%)	0.70
Prior chemotherapy – 5fu or xeloda	9(30%)	4(18%)	5(63%)	0.03
Testing center – center 1	2(7%)	2(9%)	0(0%)	
Testing center—center 2	7(23%)	5(23%)	2(25%)	0.85
Testing center—center 3	21(70%)	15(68%)	6(75%)	

### Overall Results of the In Vitro Assay

The apoptosis values for all assays are listed in Table 2. Single drugs which produced any apoptosis (>1.0 KU) in at least 50% of patient specimens were carboplatin, cisplatin, cyclophosphamide, liposomal doxorubicin, doxorubicin, epirubicin, eribulin, etoposide, ifosfamide, ixabepilone, mitoxantrone, oxaliplatin, paclitaxel, docetaxel, temozolomide, inblastine, vincristine, and vinorelbine. Drugs which produced higher levels of apoptosis (>2.0 KU) in at least 50% of specimens from patients were carboplatin, cisplatin, cyclophosphamide, paclitaxel, and docetaxel. Drugs which produced the highest apoptosis (+/- 0.57 KU) of all drugs tested in over 20% of patients were cisplatin, epirubicin, paclitaxel, temozolomide, and vinorelbine.

**Table 2:** In Vitro Drug Induced Apoptosis by Agent in all Patients.

Class	Drug	Number of Patients	Apoptosis mean KU	Apoptosis Median KU	Patients with KU>1	Patients with KU>2	Patients with highest KU +/- 0.57 KU
Alkylating Agent	Cyclophosphamide	21	2.8	2.3	17	12	3
	Ifosphamide	4	0.9	0.6	2	1	0
Anthracyclines	Liposomal doxorubicin	7	1.0	1.0	4	1	1
	Doxorubicin	21	1.8	1.6	18	6	2
	Epirubicin	22	2.2	2.0	20	10	6
	Mitoxantrone	13	1.1	1.2	9	0	1
Antimetabolites	5FU	19	0.8	0.8	8	0	0
	5FU/Methotrexate	9	1.1	0.7	4	1	1
	Gemcitabine	27	1.0	0.8	12	3	2
	Methotrexate	17	0.9	1.0	6	0	0
	Capecitabine	14	0.8	0.7	4	1	1
Combination	5FU/Cyclophosphamide/Epirubicin	6	2.5	2.8	5	4	0
	5FU/Cyclophosphamide/Methotrexate	6	1.9	1.8	4	3	0
	Carboplatin/Paclitaxel	12	2.6	2.6	10	9	5
	Carboplatin/Docetaxel	11	2.4	1.9	9	4	1
	Cyclophosphamide/ Doxorubicin	11	2.6	2.3	10	7	3
	Cyclophosphamide/ Doxorubicin/Docetaxel	6	4.1	3.5	6	5	1
	Cyclophosphamide/ Epirubicin	10	2.4	2.5	10	6	2
	Cyclophosphamide/ Paclitaxel	10	2.0	1.8	7	5	0
	Cyclophosphamide/ Docetaxel	8	3.9	2.8	7	5	3
	Capecitabine/ Vinorelbine	8	2.5	2.0	7	4	1
Other	Eribulin	10	1.2	1.3	7	1	1
	Etoposide	15	1.3	1.3	10	2	1
	Ixabepilone	18	1.4	1.2	12	4	3
	Temozolomide	3	2.3	1.9	3	1	2
Platinum	Carboplatin	22	1.7	1.6	13	9	3
	Cisplatin	17	2.8	2.4	13	12	7
	Oxaliplatin	7	1.8	1.8	6	2	0
Taxane	Nab-paclitaxel	10	0.7	0.5	3	0	0
	Paclitaxel	22	2.4	2.1	18	11	5
	Docetaxel	20	2.1	2.0	14	10	0
Vinca Alkaloid	Vinblastine	7	1.6	1.3	5	2	1
	Vincristine	9	1.3	1.3	5	1	0
	Vinorelbine	22	2.2	1.8	16	9	5

Overall the activity of cisplatin was greater than carboplatin. Overall the mean apoptosis activity for cisplatin was 1.37 KU greater than the activity of carboplatin (p = 0.0002 by Wilcoxon signed rank test which was used only in paired specimens in which both cisplatin and carboplatin were simultaneously tested). The cisplatin activity was also 1.48 KU greater than oxaliplatin (p = 0.03). In patients without any prior platinum-based therapy, cisplatin activity was 1.57 KU greater than carboplatin (p = 0.0005).

The doublet combination cyclophosphamide plus doxorubicin was more effective than doxorubicin but not cyclophosphamide. The doublet had activity 0.70 KU higher than doxorubicin alone (p = 0.04), and 1.52 KU lower than cyclophosphamide alone (p = 0.05).

The doublet combination docetaxel plus cyclophosphamide was more effective than the doublet docetaxel plus carboplatin by 1.19 KU (p = 0.04).

The doublet combination docetaxel plus cyclophosphamide (TC) had higher mean apoptosis 3.87 KU compared to doxorubicin plus cyclophosphamide (AC) 2.50 KU, but this was of borderline statistical significance (p = 0.09).

We separately evaluated the results in tumor samples from patients with triple negative breast cancer (TNBC), i.e. negative estrogen and progesterone receptors and without over-expressed Her2 (Table 3). Drugs which produced higher levels of apoptosis (>2.0 KU) in at least 50% of specimens from patients with TNBC were nab-paclitaxel, carboplatin, cyclophosphamide, doxorubicin, epirubicin, etoposide, ixabepilone, paclitaxel, docetaxel, temozolomide, vinblastine and vincristine. Since these data are based on only 6 patients, they are described only for the purpose of hypothesis generation and comparison to the data from other patients (Table 2).

**Table 3:** In Vitro Drug Induced Apoptosis in Triple Negative Breast Cancer Patients.

Class	Drug	Number of Patients	Apoptosis mean KU	Apoptosis Median KU	Patients with KU>1	Patients with KU>2	Patients with highest KU +/- 0.57 KU
Alkylating Agent	Cyclophosphamide	4	2.7	2.5	3	2	0
	Ifosphamide	1	0.0	0.0	0	0	0
Anthracyclines	Liposomal doxorubicin	2	0.6	0.6	1	0	0
	Doxorubicin	4	1.4	1.2	3	1	0
	Epirubicin	6	2.1	1.5	4	2	2
	Mitoxantrone	5	1.0	1.1	3	0	1
Antimetabolites	5FU	5	0.9	0.9	2	0	0
	5FU/Methotrexate	3	0.6	0.4	1	0	0
	Gemcitabine	6	0.8	0.6	2	1	1
	Methotrexate	4	0.9	0.9	1	0	0
	Capecitabine	4	0.5	0.7	0	0	1
Combination	5FU/Cyclophosphamide/Epirubicin	2	2.0	2.0	1	1	0
	5FU/Cyclophosphamide/Methotrexate	2	0.4	0.4	0	0	0
	Carboplatin/Paclitaxel	5	2.3	2.4	4	3	2
	Carboplatin/Docetaxel	5	2.3	2.0	4	2	1
	Cyclophosphamide/ Doxorubicin	2	2.6	2.6	2	1	0
	Cyclophosphamide/ Doxorubicin/Docetaxel	2	4.4	4.4	2	1	1
	Cyclophosphamide/ Epirubicin	3	2.6	2.9	3	2	0
	Cyclophosphamide/ Paclitaxel	3	3.2	3.9	3	2	0
	Cyclophosphamide/ Docetaxel	2	5.0	5.0	2	1	1
	Capecitabine/ Vinorelbine	3	2.5	1.2	2	1	1
Other	Eribulin	2	1.5	1.5	2	0	0
	Etoposide	5	1.2	1.2	3	1	1
	Ixabepilone	5	1.0	1.2	3	1	1
	Temozolomide	1	3.5	3.5	1	1	1
Platinum	Carboplatin	6	2.0	2.0	3	3	1
	Cisplatin	5	2.4	2.7	3	3	3
	Oxaliplatin	2	1.0	1.0	1	0	0
Taxane	Nab-paclitaxel	4	0.5	0.4	1	0	0
	Paclitaxel	6	1.9	1.9	5	2	1
	Docetaxel	6	1.8	1.4	3	3	0
Vinca Alkaloid	Vinblastine	2	2.0	2.0	2	1	0
	Vincristine	3	1.4	0.9	1	1	0
	Vinorelbine	5	2.8	1.8	4	2	2

### Patient Specific Testing in the Assay

Cisplatin was more effective than carboplatin in producing apoptosis. In 13 evaluable patients, cisplatin was more effective than carboplatin by at least 0.57 KU in 9 patients, equal in 3 patients, and less effective in 0 patients. Both drugs were ineffective in 1 patient.

Comparison of paclitaxel with docetaxel gave variable results. In 18 evaluable patients, paclitaxel was more effective than docetaxel by at least 0.57 KU in 7 patients, equal in 3 patients, less effective in 6 patients, and both agents were ineffective in 2 patients.

The doublet TC was more frequently better in the MiCK assay than AC. TC was at least 0.57 KU better than AC in 3 individual patients, better but within 0.57 KU in 3 patients, equal to AC in 1 patient, and worse than AC by at least 0.57 KU in only 1 patient.

### Physician Use of the MiCK Assay

The MiCK assay was used by physicians to help develop the treatment plan for the next therapy after the assay in 22 out of 30 patients (73.3%). When physicians used the assay, physicians changed their treatment plans in 15 out of 22 patients (68% of all patients). When the physicians utilized the assay, they utilized the best therapy in the assay in 16 out of 22 patients (73%). When physicians did not use the assay, the reasons were that the patients refused the most active drugs in the assay (4 patients), physician not wanting to use the most active drugs from the assay (2 patients), and unstated by doctor (2 patients).

When physicians used the MiCK assay, a single drug was used in place of previously planned combination therapy in 4 out of 22 patients (18.5%). Combinations were used in place of a previously planned single drug in 5 patients (23%) of patients in whom the assay was used. Only multi-source generic drugs were used in place of previously planned proprietary drugs in 9 of 22 patients (41%). In contrast, proprietary drugs only were never used in place of previously planned generic drugs (0%).

Physicians used the MiCK assay results alone or with additional agents in 22 of 30 patients. When the physicians used the MiCK assay, in 12 out of 22 patients only the MiCK assay was used (55%). Drugs in the MiCK assay were combined with hormonal therapy in 6 of 22 patients (27%), and the MiCK Assay was used in combination with biotherapy (trastuzumab or bevacizumab) in 6 out of 22 patients (27%). In the 8 patients in whom physicians did not use the assay, it was based on physician preference for a particular chemotherapy regardless of the assay results or patient preference for a chemotherapy different from the assay results.

### Correlation of Outcomes with Use of the MiCK Assay

The frequency of complete (CR) or partial response rates (PR) in patients according to whether or not the MiCK assay was used are presented in Table 4. In contrast to the 38.1% response rate obtained in 22 patients whose physician used the MiCK assay, there were no responses seen in the 8 patients whose physician did not use the MiCK Assay (p = 0.04).

**Table 4:** Correlation of Response with use of the Mick Assay.

MICK ASSAY USED	CR+PR	STABLE+PROGRESSION
YES	8 (38.1%)	13 (61.9%)
NO	0 (0%)	8 (100%)
	P = 0.04	

CR, complete response; PR, partial response.

In addition, CR plus PR plus stable responses (disease control) were more frequently observed if the physician used the MiCK assay (Table 5). If the MiCK assay was used with the patient, 17/21 patients (81%) had disease control compared to 2 of 8 patients (25%) in whom the MiCK assay was not used. (p<0.01) Note that 1 patient was in evaluable for tumor response.

**Table 5:** Correlation of Response with use of the Mick Assay.

MICK ASSAY USED	CR+PR+STABLE	PROGRESSION
YES	17 (81%)	4 (19%)
NO	2 (25%)	6 (75%)
	P<0.01	

**Fig 2:**
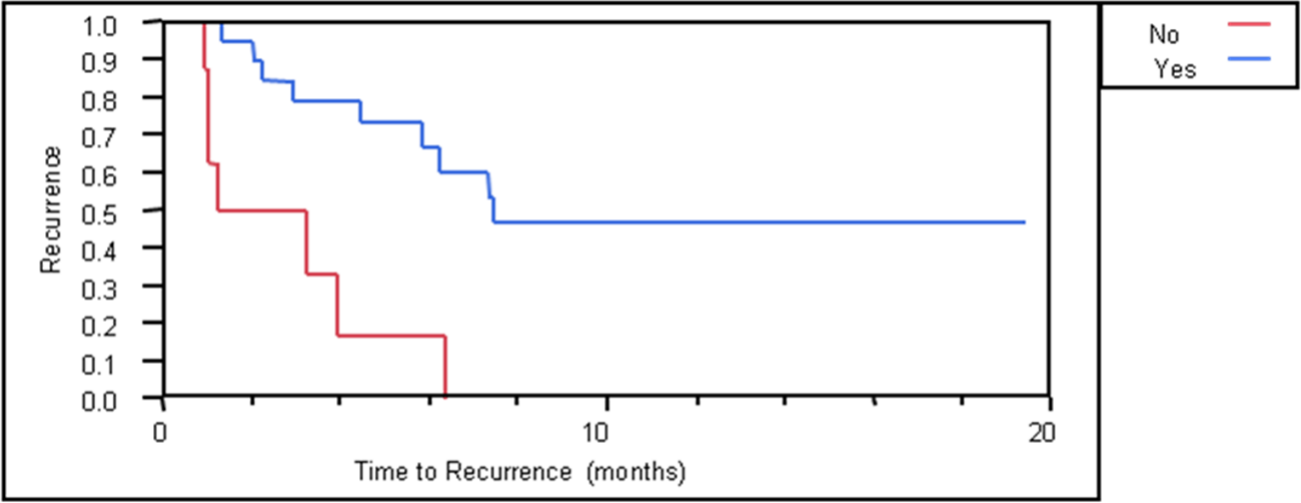
Time to recurrence in patients with recurrent or metastatic breast cancer. Blue curve, patients whose physician used the MiCK assay. Red curve, patients whose physician did not use the MiCK assay.

The time-to-recurrence was compared according to whether or not physicians used the MiCK assay (Fig 2). The median time-to-recurrence if the MiCK assay was used was 7.4 months, compared to only 2.2 months if the physician did not use the MiCK assay (p<0.01).

**Fig 3:**
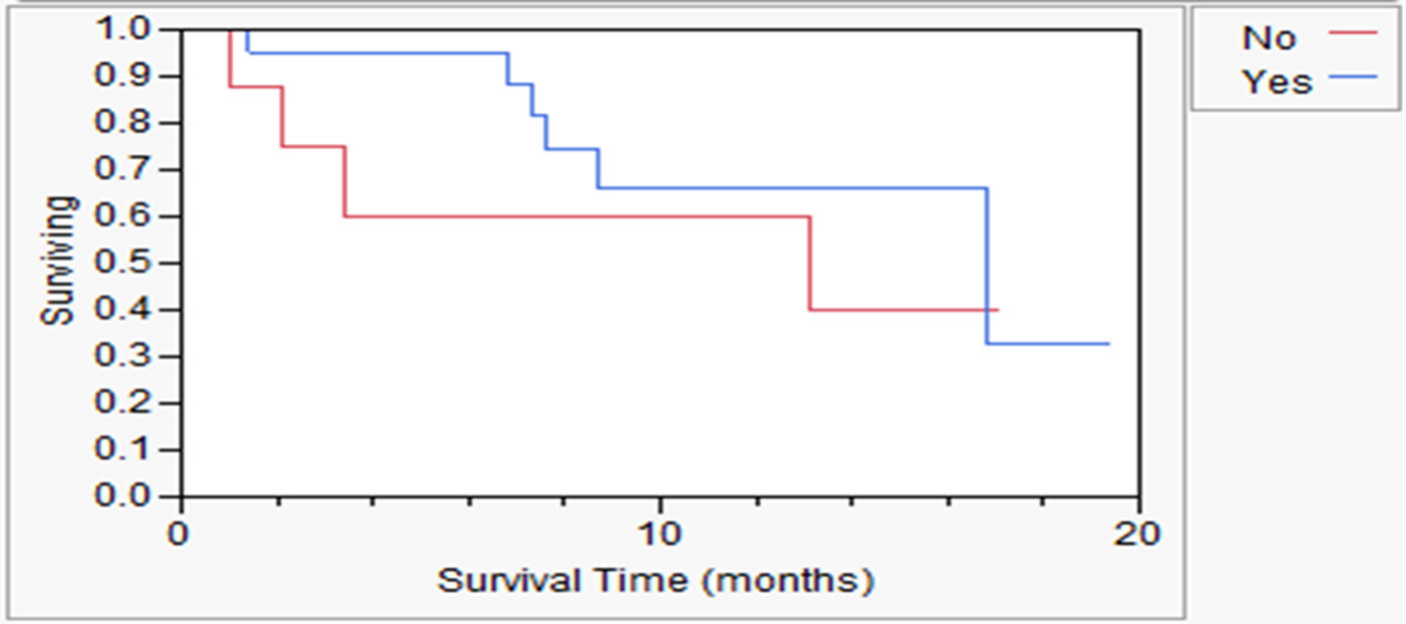
Overall survival in patients with recurrent or metastatic breast cancer. Blue curve, patients whose physician used the MiCK assay results for next treatment after assay. Red curve, patients whose physician did not use the MiCK assay results for next treatment after assay.

The overall survival of patients was evaluated according to the physician use of the MiCK assay to determine the next chemotherapy after the assay (Fig 3). There was a trend toward improved survival if patients used the MiCK assay (16.8 months) compared to patients whose physician did not use the MiCK assay (13.1 months). This difference is not yet statistically significant (p = 0.33). However, in some instances where the physician did not use the MiCK assay for the first chemotherapy after assay results were obtained, the physician used the assay for subsequent lines of chemotherapy (reducing the potential difference between survival in early users versus those who did not use the MiCK assay initially after testing).

## Discussion

Apoptosis has been widely studied and its application to clinical care has been explored without definitive positive results. This study indicates that this drug induced apoptosis assay can be successfully used in tumor specimens from patients with recurrent or metastatic breast cancer. The drug induced apoptosis results were unique individually and could not be predicted for individual patients without performing the assay.

The overall results of the assay were informative about the relative degrees of activity of different classes of drugs, and individual drugs within each class in this previously treated patient population with recurrent or metastatic breast cancer. This information has not been previously demonstrated. Active drugs could be identified and ranked, combinations could be compared to single drugs, and combinations could be compared to other combinations. Many active drugs are approved for use in patients with metastatic breast cancer so an assay that can guide the best choice of these drugs which improves clinical responses will be important in facilitating value based patient care. This assay can also help further drug development in the future. In evaluating new drugs, the average activity of a new agent can be compared in the assay with other approved drugs, the frequency of patients in which a new drug is superior to other therapies can be identified and quantified, and combinations of a new drug with other drugs can be compared.

A particular challenge in breast oncology is determining the optimal treatment for patients with triple negative breast (TNB) cancer [16]. The assay performed well in identifying agents producing the highest apoptosis in such patients (7 TNB patients of the 30 total patients tested) although the numbers are small. The best regimens in TNBC included platinum drugs in 7 of 7 samples, anthracyclines in 4/7 samples, taxanes in 6/7 samples, cyclophosphamide in 2/7 samples, plus other drugs in selected patients. These drugs included vinorelbine, gemcitabine, temozolomide, etoposide, ixabepilone, and capecitabine. These results suggest that the assay may help developing new treatment strategies, including novel drugs, and individualizing care for TNBC patients.

The assay also has value in comparing within-class drugs. Although genomic assays can only predict which class of drugs might have activity in an individual patient, based on gene or gene product levels, the MiCK assay can identify which drug in a class is superior. In the data presented, average activity of cisplatin was greater than carboplatin and greater than oxaliplatin.

The greater value of the assay is in identifying on a patient by patient basis which drugs are superior, a clinical necessity where sensitivities, especially in previously treated patients cannot be reliably predicted and the current standard approach of ensuring sequential therapy after an anthracycline and a taxane is capecitabine and then eribulin or ixabepilone, may not be the most active sequential therapy for every patients. For example, cisplatin was always more active than, or equally active to carboplatin in each patient. However, paclitaxel was more active than docetaxel in some patients, equally active in some patients, and less active in others. Clinical information could not predict these results in an individual. This suggests that our current regimens might be substantially more effective if the most active drug or combination were to be utilized, and the best within-class agent would be selected rather than the agents giving the best overall results from large phase III breast cancer clinical trial populations. Indeed, the goal of all personalized therapy is to understand unique differences in similar stage and tumor types that predict better outcomes, the MiCK assay as it continues to be validated may play an important role in helping physicians achieve individualized therapeutics with their patients and improved outcomes.

In vitro results also identified some drugs with unanticipated activity in breast cancer. Temozolomide and etoposide are not conventionally used in palliative care in breast cancer, but results from the assay suggest that drugs such as these, as well as others that could be tested in future applications, might identify treatments with value to individual patients. The MiCK assay may identify specific drugs that work for a specific patient even though their use would not be effective for in a large population of patients and would thus fail conventional testing in clinical trials. Such drugs with activity in only a few patients might succeed in clinical or registry trials if the MiCK assay were used as a companion diagnostic to select patients with a high likelihood of response.

When physicians received the results of the drug induced apoptosis assay, there was a high usage rate by oncologists. Comparison of the patients whose physicians used the MiCK assay with those whose physicians did not use the assay was similar. However, there was a non-significant trend to later line of therapy to be used next in patients in whom the physician did not use the MiCK assay (4^th^ line median) versus earlier line of therapy to be used next in patients in whom the physician did use the MiCK assay (2^nd^ line median), p = 0.07. This was not due to a difference in performance status. This indicates that there might have been a selection bias by the physicians in tending to the MiCK assay more often in earlier lines of therapy compared to selecting therapy in later lines of therapy. This bias, or perhaps even accidental bias (due to the non-randomized nature of this utility trial) should be eliminated by subsequent randomized clinical trials which are planned.

Furthermore, translation of the preclinical assay science to the clinic was associated with an improvement in response rate (CR plus PR), disease control rate (CR plus PR plus stable), and time-to-recurrence if the physician used the results to develop the treatment plan, but not if the physicians did not use the assay results. This indicates that the assay is predictive, not prognostic. A trend to increased survival was seen, but is somewhat confounded by physicians’ subsequent use of the MiCK assay for follow-up therapies (crossover use) in patients in whom the physician initially did not use the assay for the first treatment after the assay was performed and the small pilot sample size. There is a trend toward increased survival in the group when the physician initially used the assay (Fig 3). While statistical comparisons were made between the patients whose doctor used the assay versus those who did not use the assay, and were found to be significant, it should be noted that this was not a randomized trial. The 2 groups might have been biased. However, these descriptive comparisons justify a larger randomized trial, which is planned, and help to give metrics on which to base the power calculations and enrollment requirements of such a study. Such a study should have only one method for evaluation of tumor response such as RECIST and verification of response by an external review. Such a study should also accrue enough patients to be certain that the assay and its applicability can be understood in the major subtypes of breast cancer.

Notably, the assay was also able to identify patients in whom no drug produced significant apoptosis (all KU values under 1.0) (this applied to 30 patients). Use of the assay in such patients helped physicians to transition patients into palliative care, hospice, or phase I clinical trials programs. This may reduce futile chemotherapy, unnecessary suffering and futile toxicity management costs at end of life.

Therapy of TNBC has been challenging with poor prognosis [17]. Response rates to chemotherapy are usually less than 50% [18]. Analysis of the TNBC subset in this study indicates that the assay can be used in such patients, and can help identify drugs with higher levels of apoptosis as a basis for future drug trials.

These results of the MiCK drug-induced apoptosis assay are an improvement over the results of prior chemoresistance assays [1]. The MiCK drug induced apoptosis assay is much different since it relies on a different scientific principle of light physics (optical density based on Mie light scattering effects) rather than biochemistry or cellular dye transport. Also, the MiCK assay uses only purified cancer cells rather than mixed cells, requires no cancer cell growth, has results available in 48–72 hours, measures results every 5 minutes over 48 hours (rather than one determination at the end of most other types of assays).

This study indicates that when physicians receive the MiCK assay results in patients with breast cancer, they frequently use the results to determine therapy (73%). Use of the assay correlated with outcome benefits to the patients.

These results provide supportive data for future trials to determine the impact of the MiCK assay in patients receiving neoadjuvant and adjuvant therapy, as well as further trials to determine the impact on different lines of palliative chemotherapy in patients with recurrent and metastatic breast cancer. This reported utility trial also provides evidence on how the MiCK assay can help oncologists to plan treatment decisions for patients with metastatic breast cancer. These results from a good laboratory phase II study can be the basis for a future larger prospective multicenter study to more definitively establish the value of the assay in breast cancer, such as the studies which have been done previously in acute myelocytic leukemia [2] and ovarian cancer [3].

## Supporting Information

S1 CONSORT Checklist (DOCX)

S1 Protocol (DOC)
