# Supplementary material for: Application of a Drug-Induced Apoptosis Assay to Identify Treatment Strategies in Recurrent or Metastatic Breast Cancer
Source: PLoS One. 2015 May 29;10(5):e0122609. doi: 10.1371/journal.pone.0122609 (PMC4449169; doi:10.1371/journal.pone.0122609)
Supplement: S1 Protocol — (DOC) [file pone.0122609.s002.doc]

Correlation of the Microculture Kinetic (MiCK) Apoptosis Test Results with Drug Treatment Results in Cancer Patients

PRINCIPAL INVESTIGATOR:

CO-PRINCIPAL INVESTIGATOR:

MEDICAL MONITOR: Cary Presant, MD

SPONSOR: DiaTech Oncology, LLC, Garry Latimer, CEO

-----------------------------------------------------------

**1. 0 Background and Rationale:**

Identification of those patients with cancer who will or will not respond to a specific chemotherapy is important for making decisions regarding chemotherapy regimens as well as alternative management approaches. A laboratory test that could help to determine the sensitivity of an individual patient’s tumor cells to specific chemotherapeutic agents would be valuable in choosing the optimal chemotherapy regimen for that patient with an expectation of increasing the response rate to the therapy. Several types of *in vitro* assays that measure tumor cell survival following exposure to cytotoxic agents have been evaluated for their ability to predict chemotherapy outcomes. As a group, these assays are referred to as drug resistance assays. In a resistance assay, the surviving tumor cells can be detected directly by their exclusion or metabolism of specific dyes. Alternatively, since some of tumor cells are proliferating, their survival can be detected by measurement of DNA synthesis by radiolabeled precursor incorporation or demonstration of clonogenic potential by growth into colonies in semi-solid culture medium. In several clinical studies, these assays were useful in detecting drug resistance and in predicting a poor prognosis for cancer patients. However, these resistance assays cannot detect sensitivity of an individual patient’s tumor cells to a specific drug. Therefore, new methods determining drug-sensitivity of the tumor cells of an individual patient and, thus, capable of both predicting a positive treatment outcome and guiding chemotherapy, would be of significant value.

Recently, an automated microculture kinetic (MiCK) assay for measuring drug induced apoptosis in tumor cells has been developed^1-4^. Apoptosis is a distinct mode of cell death which occurs under physiological conditions and yet can be induced in malignant cells by chemical and physical factors including antitumor drugs^5-7^. During the last decade, it has been recognized that chemotherapeutic agents exert their antitumor activity by triggering apoptosis in susceptible tumor cells^8-17^. This implies that the MiCK assay for apoptosis provides a mechanism-based approach to studying effects of cytotoxic agents on tumor cells. Unlike “resistance” assays that measure a fraction of cells surviving drug exposure, the MiCK assay measures a fraction of tumor cells killed by a chemotherapeutic agent via mechanism of apoptosis. Therefore the MiCK assay determines drug sensitivity, rather than resistance. Recently the MiCK assay has been shown to predict complete remission rate and survival in acute myeloid leukemia patients better than clinical criteria did^18-20^. In a limited study, the MiCK assay has been used to direct chemotherapy of the leukemia patients ^21^.

The MiCK assay has also been used to study drug-induced apoptosis in solid tumors, including neuroblastoma and colon adenocarcinoma cell lines^22-23^. More recent data accumulated by DiaTech has demonstrated that the MiCK assay can detect drug induced apoptosis in primary cultures of tumor cells isolated from patients with ovarian carcinoma, gastric carcinoma, metastatic breast cancer and high grade soft tissue sarcoma. The purpose of this study is to correlate the results of the MICK assay with short- and long-term results of treatments in cancer patients and evaluate the role of the MiCK assay in guiding chemotherapy of cancer patients.

1. **Study Objectives:**
   1. To correlate the MiCK assay results with objective response rates, symptom response rates, time to progression and survival of cancer patients treated with chemotherapy.
   2. To evaluate the ability of the MiCK assay to guide chemotherapy of cancer patients, with emphasis on patients failing primary treatment, patients with unknown primary tumors, and patients with tumors difficult to treat such as carcinoma of lung.
   3. To determine healthcare costs, and resource utilization when MiCK assay is used in treatment decision.
2. **Patient Population:**
   1. *Inclusion criteria:*
      1. Patients with pathological diagnoses of cancer or leukemia
      2. Patients must have tumor which is accessible for biopsy and agree to undergo tumor biopsy, or drainage of malignant effusion, and the specimen must be submitted for MiCK assay.
      3. Patients for whom chemotherapy is planned.

- 1. *Exclusion criteria:*
     1. Patients with symptomatic/uncontrolled parenchymal brain or meningeal metastasis and tumors not accessible for biopsy.
     2. Patients who are pregnant.

Pregnancy. During the course of the study, all patients of childbearing potential should be instructed to contact the treating physician if they suspect they might have conceived a child; for females, a missing or late menstrual period should be reported to the treating physician. If pregnancy is confirmed by a pregnancy test, the patient must not receive chemotherapy in this study and must not be enrolled into the study or, if already enrolled, must be withdrawn from the study. If a male patient is suspected of having fathered a child while on the study, the pregnant female partner must be notified and counseled regarding the risk to the fetus. Pregnancy during the course of this study will be reported to the Principal Investigator as a serious adverse event. Women of child bearing potential are defined to include any female who has experienced menarche and has not undergone successful surgical sterilization (hysterectomy, bilateral tubal ligation, or bilateral oophorectomy) or is not post-menopausal (defined as amenorrhea for more than 12 consecutive months); these includes also females using oral, implanted, or injectable contraceptive hormones, mechanical devices, or barrier methods to prevent pregnancy.

1. **Treatment Plan:**

4.1 The treating oncologist will decide what chemotherapy to use with each patient. The treating physician will be fully informed of the results of the MICK assay.

4.2 Tumor response, patient symptom response, time to progression on each chemotherapy regimen, and overall survival will be reported and correlated with MICK assay results. Cost of care, and utilization of resources will also be measured and de-identified claims data will be used to determine some of these parameters. Physicians will provide information on the intended choice of chemotherapy without the assay results, and the choice after assay results.

**5.0 Definition of Clinical Response**

5.1 All patients will be evaluated for clinical response to chemotherapy as per standard protocol established for a specific malignancy.

5.1.1 Complete Remission CR is defined as total disappearance of clinically and radio logically detectable disease.

5.1.2 Partial Response PR is defined as at least 50% reduction of all measurable tumor lesions as measured by the sum of products of the perpendicular diameters of the greatest dimensions of measurable lesions, with no new lesions appearing; or by a greater than 30% reducion in the sum of diameters or unidimensionally measured tumors with no new lesions appearing, or by >50% reduction in tumor markers.

5.1.3 Stable Disease SD is defined as no CR, no PR and no PD 5.1.4 Progressive Disease PD is defined as appearance of any new tumor lesions or increase of 50% or more in the sum of products of the perpendicular diameters of the greatest dimensions of measurable existing lesions, or increase of 20% in the sum of diameters of unidimensional lesions, or 100% increase in tumor marker

- - 1. **Time to progression TTP. The time from assay until progressive disease PD or death**
    2. **Overall Survival OS. Time from assay until death**
    3. **Symptom Response is evaluated by the physician based upon patient history and examination, and will be listed as improved, stable, or worse.**

**6.0 Definition of the drug response in the MiCK assay.**

6.1 In the MiCK assay, the extent of drug-induced apoptosis is measured in Kinetic Units (KU) on a scale from 0 to 16 (Blood, 1998). Apoptotic responses in KU will be calculated for each dose of a tested drug or drug combination. The maximal numerical response induced by a drug will be considered the “best apoptotic response” to the drug.

6.2 The “best apoptotic response” to the drug will be compared to the clinical response to the drug to establish a numerical value of the *in vitro* response discriminating drug-sensitive and drug-insensitive tumors (establishing a numerical cut-off of sensitivity).

6.3 All response data will be given to the treating oncologist who will retain full choice of treatments.

6.4 Drug selection for testing with the MiCK assay against tumor cells will be based on the previous chemotherapy history of an individual patient with consideration of the future treatment plans. The drugs will be selected from a compendium of agents recommended for the treatment of the patient’s malignancy and drugs suggested by the treating oncologist.

**7.0 Statistics**

- 1. We will correlate tumor response, symptom response, TTP and OS with drug therapy with drug response in MICK assay for first chemo regimen after assay, and subsequent regimens.
  2. We will compare tumor response, symptom response, TTP and OS for patients treated with drugs showing “best apoptotic response”, drugs showing any apoptotic response (>= 1.0 KU) and drugs showing no apoptotic response (0-<1.0 KU).
  3. We will evaluate costs of care, utilization of resources (e.g. hospitalization, ER visits), and changes in chemotherapy treatment plan.

**8.0 Specimen collection, purification of tumor cells and sensitivity testing**

8.1 Specimen collection must be performed as per related DiaTech Standard Operating Procedures (SOP).

- - 1. Collection of a Solid Tumor Biopsy Specimen is performed under sterile conditions, using excision biopsy technique, to obtain at least 2 cm^3^ of viable tumor tissue. The more viable tumor tissue is submitted for the study, the more chemotherapeutic agents can be tested against the tumor cells. Effusion specimen (peritoneal fluid, pleural fluid and effusions from other anatomical sites) should be collected in a commercial sterile container/bag with added Sodium Heparin (10 U/ml) to prevent clotting. The sample size should not be less than 500 ml. The specimen should not be fixed, or frozen.
    2. Transportation: seal the transport tube/container/bag tightly. Label specimen with institution, patient name, date and time of collection, and anatomical site of collection. Place the transport tube/container/bag on ice pack (blue ice). Ice pack must be frozen before placing it to the container. Fill out the Study Requisition form and include with the transportation container.
    3. Place the 50 mL tubes or a container inside the zip-lock bag and seal. Place specimen and Cold Pak (blue ice) in a transport box. Place completed patient information forms and specimen transport box into FedEx plastic “Diagnostic Specimen Envelope” and seal. Complete and affix the FedEx Airbill to the outside of the Diagnostic Specimen Envelope. Be sure that the airbill is marked “FedEx Priority Overnight” delivery. Put $5 value on the FedEx Airbill. Indicate “Human tissue for diagnostic studies” in the appropriate section of the FedEx Airbill. Call DiaTech Oncology at (514)-398-5174 (Mathieu Perree, lab manager) or (514)-398-5154 (general lab) with the Fed Ex Tracking Number. DiaTech must receive the specimen within 24 hours of collection. Specimens sent on Friday must be marked for Saturday delivery.
  1. Tumor cell purification and their chemosensitivity testing will be performed as per related DiaTech SOPs.
     1. Tissue biopsy and effusion specimens will be treated to obtain a suspension containing single tumor cells and/or small cell aggregates composed of 2-20 tumor cells. Using gradient centrifugation, red blood cell lysing, cell strainers, magnetic beads and other appropriate techniques, the tumor cell suspension will be enriched to at least 80% purity and no less than 90% viability. Immunocytochemical stains or, when applicable, flow cytometry will be used to confirm the presence of specific tumor markers on purified cells After purification, selected chemotherapeutic agents will be tested against purified tumor cells in the MiCK assay.
     2. The purified tumor cells will be suspended in culture medium and plated in 96-well microtiter plates. Multiple concentrations of chemotherapeutic agents will be achieved by adding each respective agent to wells in 5 μL aliquots The ranges of final drug concentrations will be based on reports of pharmacokinetic studies of the drugs and their active metabolites in patients. Data processing and quantification of drug-induced apoptosis will be performed by a proprietary ProApoTest^TM^ software. The extent of apoptosis will be determined and expressed as kinetic units (KU) of apoptosis.

1. **Specimen’s left over**

9.1 If a specimen contains more tumor cells than needed for testing their sensitivity to the drugs specified by the patient’s oncologist, an excess of the tumor cells may be considered for use for other research studies conducted by DiaTech Oncology. Patients will be asked for their permission to use specimen’s left over for research purposes by signing the following release included in the requisition form: “*You are participating in the Research Program which may result in improvements of the treatment outcome for cancer patients. There is no direct benefit for you at present time. Your physician is submitting to DiaTech a specimen containing your tumor cells. At DiaTech, we purify the tumor cells, count them, check their viability, and store them in the DiaTech Tissue Bank. All these procedures are performed at no cost to you or your family. Your cells may be used for the chemosensitivity testing for research purposes or in other studies. Results of the research may be published, or used commercially in the area of new anti-cancer drug or treatment protocol development. To assure your privacy, should the results of the studies be published, you will be referred to only by number. Your signature below indicates that you agree to these terms*”.

If a patient refuses to sign the above release, the specimen’s left over will be decontaminated using 10% formaldehyde for 24h and discarded as per related SOP. If a patient grants his/her permission for use on the specimen’s left over in a future research, an excess of the tumor cells will be frozen and stored in liquid nitrogen indefinitely.

9.2 Specimen’s left over may be used to study anti-tumor effects of chemotherapeutic drugs or drug combinations, to study mechanisms of drug resistance, to correlate phenotypic features of the tumor cells with their drug sensitivity profile.

1. Plan of communication between ***DiaTech oncology lab director,*** Principal Investigator and co-Principal Investigator.

To insure proper communication the following communication means will be used: FedEx delivery of the printed materials, Phone & Fax, E-mail, Video-conferencing

- 1. At the time of submission of the patient’s specimen for the study, relevant clinical information will be submitted to DiaTech in the form of a study requisition form prepared by the ***referring oncologist*** (or designated staff nurse) using FedEx courier service.
  2. Upon receiving the specimen and requisition form and after purification of tumor cells from the specimen, ***DiaTech personnel*** will place a telephone call to the referring oncologist (co-PI) to discuss the case.
  3. Upon completion of the patient’s tumor drug sensitivity testing, results will be scored and introduced to the study data base. ***The DiaTech Oncology lab director*** will issue a study report and it will be faxed to the referring oncologist no later than 96h after receiving the specimen. After faxing the report, ***DiaTech pesronnel*** will call the referring oncologist to discuss the results.
  4. After each 2 cycles of therapy, or at other intervals, the patient’s treatment response will be evaluated by the referring oncologists (co-PI). A study Response Evaluation Form will be filled out and faxed to DiaTech to be included in the study data base.
  5. DiaTech Study coordinator (Mr. Garry Latimer 615 377 9668) will contact each site co-PI or a designated nurse monthly to assure proper supply of transportation containers and to address administrative issues.

.

**RESEARCH SUBJECT INFORMATION AND CONSENT FORM**

**TITLE:**  Correlation of the Microculture Kinetic (MiCK) Apoptosis Test Results with Drug Treatment Results in Cancer Patients

**PROTOCOL NO.:** None

WIRB® Protocol #20082132

**SPONSOR:** DiaTech Oncology, LLC

Brentwood, Tennessee

United States

**INVESTIGATOR:**  Name and Degree

Address

City, State, Zip

Country

**SITE(S):** Location

Address

City, State, Zip

Country

**STUDY-RELATED**

**PHONE NUMBER(S):**  Name and Degree

Phone number

This consent form may contain words that you do not understand. Please ask the study doctor or the study staff to explain any words or information that you do not clearly understand. You may take home an unsigned copy of this consent form to think about or discuss with family or friends before making your decision.

In this consent form, “you” always refers to the subject. If you are a legally authorized representative, please remember that “you” refers to the study subject.

**SOURCE OF FUNDING**

DiaTech Oncology is the sponsor of this study.

**GENERAL**

You have been asked to participate in a clinical research study. You have been told also that you have the option not to participate. This study is being carried out under the sponsorship of the DiaTech Oncology Corporation. DiaTech is a private company doing chemosensitivity testing for patients and physicians. This research study will be listed with the National Cancer Institute (NCI). DiaTech Oncology is setting up a clinical study to test the ability of an experimental technology called the Microculture Kinetic (MiCK) assay (test) to help predict treatment outcome and to help decide chemotherapy for cancer patients.

**HOW MANY SUBJECTS WILL TAKE PART IN THE STUDY?**

Up to 2000 subjects will participate in this study.

**WHAT IS INVOLVED IN THE STUDY?**

**Medical Tests:**

You will have an evaluation before the start of the study. This evaluation will include:

- A physical examination which may include pelvic examination
- Lab tests to determine blood counts
- An assessment of liver and kidney status through blood studies.

Any detectable tumors will be measured by examination, radiology and blood tests. A biopsy of your cancer or body fluids with cancer cells will be sent to DiaTech for the MiCK assay. Results will be sent to your doctor who will decide with you which drugs will be used to treat your cancer. Data from follow up evaluations after chemotherapy has been started will be sent to the DiaTech for analysis as a part of the study to determine how well the MiCK assay has predicted your response to the chemotherapy. Information on how much my treatment has cost may be evaluated.

**The parts of this study that are being done solely for this study.**

Treatments and evaluations are standard care for your cancer. The only parts of this study that are being done solely for this study are comparisons of how well you respond to the chemotherapy compared to the results of the MiCK laboratory test and how much the test can help to reduce the costs of care including how much you actually have to spend on your care.

**HOW LONG WILL I BE IN THE STUDY?**

At least 1 year.

**WHAT ARE THE RISKS OF THE STUDY?**

There are no medical risks from being in this study. There is a risk of loss of confidentiality from your research data.

**NEW FINDINGS**

You will be told about any new information that might change your decision to be in this study. You may be asked to sign a revised consent form if this occurs.

**Possible benefits of this study.**

This study may allow physicians to select the best chemotherapy drug for cancer patients in the future. However, there is no direct benefit to you from being in this study.

**PAYMENT FOR PARTICIPATION**

You will not be paid for taking part in this study.

**ALTERNATIVES**

Participation in this study is voluntary. Your alternative is to not be in this study.

**COMPENSATION FOR INJURY**

DiaTech Oncology is not offering any compensation for injury that may occur as a result of the treatments and evaluations.

**PRIVACY AND CONFIDENTIALITY**

You have the right to privacy. All information will be held confidential and will not be released without your written permission to the extent permitted by law.

Efforts will be made to keep your name and personal information confidential. Absolute confidentiality cannot be guaranteed, however confidentiality will be maintained to the extent permitted by local, state, and federal law.

Your personal health information will be used and disclosed to DiaTech personnel for this research study. A decision to participate in this study means that you agree to the use and disclosure of your personal health information only for the purposes explained in this consent form.

During this study the study team may use the following health information from you:

- Hospital records
- Doctor’s office records
- Test results
- Exams or procedures conducted for this study as described in this consent form.
- Data about the costs of your care, sent from your health plan.

Only the researchers and hospital staff conducting this study will have access to your personal health information as necessary to conduct this research study.

Your personal health information may also be disclosed to representatives of the study sponsor, Diatech Oncology Company, the U.S. Food and Drug Administration (FDA) or other regulatory agencies, or Western Institutional Review Board® (WIRB®) to monitor the conduct of the study. There is the potential of further disclosure of your personal health information by these individuals or entities such that your information is no longer subject to protection under federal regulations governing the privacy of health information. This research may result in scientific presentations and publications, but precautions will be taken to make sure you cannot be identified in any way.

By signing this consent form, you authorize the use of your personal health information until the end of this study. You have the right to revoke your authorization to use your personal health information. The revocation must be in writing and sent to your medical oncologist. If you revoke your authorization it will not apply to prior uses or disclosures of your personal health information made in accordance with the purposes explained in this consent form.

If you refuse to provide authorization to use and disclose your personal health information for this study, the study doctor may refuse to include you as a subject in this study.

Your right to access your health information used and disclosed for the study may be restricted as long as the research is in progress; however, your right to access will be reinstated, upon completion of the research study.

**VOLUNTARY PARTICIPATION/WITHDRAWAL**

Your participation in this study is voluntary. You may decide not to participate or you may leave the study at any time. Your decision will not result in any penalty or loss of benefits to which you are entitled.

Your participation in this study may be stopped at any time by the study doctor or the sponsor without your consent for any of the following reasons:

- if it is in your best interest;
- you do not later consent to any future changes that may be made in the study plan;
- or for any other reason.

**QUESTIONS**

Contact at for any of the following reasons:

- if you have any questions about your participation in this study,
- if at any time you feel you have had a research-related injury, or
- if you have questions, concerns or complaints about the research

If you have questions about your rights as a research subject or if you have questions, concerns or complaints about the research, you may contact:

Western Institutional Review Board® (WIRB®)

3535 Seventh Avenue, SW

Olympia, Washington 98502

Telephone: 1-800-562-4789 or 360-252-2500

E-mail: Help@wirb.com.

WIRB is a group of people who perform independent review of research.

WIRB will not be able to answer some study-specific questions, such as questions about appointment times. However, you may contact WIRB if the research staff cannot be reached or if you wish to talk to someone other than the research staff.

Do not sign this consent form unless you have had a chance to ask questions and have received satisfactory answers to all of your questions.

**Overview:**

**Detailed Information:**

**Making Your Choices About Future Research**

Please read each sentence below and think about your choice. After reading each sentence initial your choice. **No matter what you decide to do, it will not affect your care.** If you have any questions, please talk to your study doctor, study nurse or your healthcare provider.

1. Do you give permission for your specimens, if still available after this research study is completed, to be used in future research to learn about, prevent, or treat cancer?

Yes No

1. Do you give permission for your specimens, if still available after this research study is completed, to be used in future research to learn about, prevent or treat health problems other than cancer (for example: diabetes, Alzheimer’s disease, or heart disease)?

Yes No

If you agree to be in this study, you will receive a signed and dated copy of this consent form for your records.

**CONSENT**

I have read the information in this consent form. All my questions about the study and my (my child’s) participation in it have been answered. I freely consent to be (to allow my child to be) in this research study.

I authorize the use and disclosure of my (my child’s) health information to the parties listed in the authorization section of this consent for the purposes described above.

By signing this consent form, I have not given up any of my/my child’s legal rights.

***Consent and Assent Instructions:***

*Consent: Subjects 18 years and older must sign on the subject line below*

*For subjects under 18, consent is provided by the parent or guardian*

*Assent: Is not required for subjects 12 years and younger*

*Verbal assent is required for subjects ages 13 through 17 years using the Assent section below.*

Subject name:

**CONSENT SIGNATURE**

Subject signature (18 years and older): Date:

Signature of Parent or Guardian: Date:

(when applicable)

Signature of Person Conducting

Informed Consent Discussion: Date:

Signature of Attending

Study Doctor/Study Nurse: Date:

(if different from above)

**ASSENT SECTION**

Statement of person conducting assent discussion:

1. I have explained all aspects of the research to the subject to the best of his or her ability to understand.
2. I have answered all the questions of the subject relating to this research.
3. The subject agrees to be in the research.
4. I believe the subject’s decision to enroll is voluntary.
5. The study doctor and study staff agree to respect the subject’s physical or emotional dissent at any time during this research when that dissent pertains to anything being done solely for the purpose of this research.

________________________________________ __________________

Signature of Person Conducting Date

Assent Discussion

Statement of Parent or Guardian:

My child appears to understand the research to the best of his or her ability and has agreed to participate.

________________________________________ __________________

Signature of Parent or Guardian Date

APPENDIX B

| **REQUSITION FORM FOR THE DIATECH TUMOR STUDY**  **FAX COMPLETED REQUSITION TO 514-398-4939** | | | | |
| --- | --- | --- | --- | --- |
| Patient Name | | | Institution/Hospital | |
| Patient’s Sex £M £F | Patient D.O.B.  (mm/dd/yyyy) | | Referring Physician | |
| Patient’s Unique Identifier Number | | | Referring Physician Phone/Fax# | |
| Sender Specimen # | | ICD9 code: | Send Report To: |  |
| Specimen Type £Whole Blood £Bone Marrow £CSF  £Lymph Node £Other (specify): | | | Date Time collected □ am  □ pm | |
| **PATIENT’S DIAGNOSIS** | | | | |
| 1. Original diagnosis (please specify subtype of the malignancy and staging): | | | | |
| Date the original diagnosis was made: | | | | |
| Is this the first presentation or relapse? | | | | |
| If prior systemic therapy was used, please describe all drugs previously used: | | | | |
| a. chemotherapeutic agents used (if known): | | | | |
| b. patient’s response to the treatment (CR, PR, No response): | | | | |
| c. date of the most recent course of chemotherapy: | | | | |
| Drugs to be considered for use and to be tested:  Preferred:  Others:  Do not need to test:   | **COMPLETED BILLING INFORMATION** | | | | | | | | --- | --- | --- | --- | --- | --- | --- | | Responsible Party | | Responsible Party S.S. # | | | | | | Responsible Party Billing Address | | City | State/Province | | | Zip Code | | Responsible Party Telephone Number | | Relationship to Patient  □ Self □ Spouse □ Other: | | | Patient Birth Day | | | Responsible Party Place of Employment | Employment Address | | | Business Telephone | | | | Type of billing □ Visa □ Master Card  Number □□□□ □□□□ □□□□ □□□□ Exp. Date □□/□□ | | | | | | | | **INFORMED CONSENT FORM** | | | | | | | | **To the patient.** Since you were diagnosed with a cancer, your physician has ordered the MiCK assay to determine the sensitivity of your tumor cells to chemotherapy drugs. Specimens submitted for the MiCK assay may contain more cells than required for the test. The tumors cells not used for the MiCK assay are stored under special conditions at low temperatures. They may be used in the future for research purposes. Results of the research may be published, or used commercially in the area of new anti-cancer drug development. To assure your privacy, should the results of the studies be published, you will be referred to only by number. Your signature below indicates that you agree to these terms.  Patient Signature Date Signature of witness(es) (when applicable) Date | | | | | | | | | | | |

**REFERENCES**

1. Kravtsov V. A novel microculture kinetic assay (MiCK assay) for malignant cell growth and chemosensitivity. Eur J Cancer,30A,10,1564-70,1994
2. Kravtsov V, Fabian I. Automated monitoring of apoptosis in suspension cell cultures. Lab Invest,74,2,557-70,1996.
3. Kravtsov V, Greer J,Whitlock J, Koury M Use of the microculture kinetic (MiCK) assay of apoptosis to determine chemosensitivities of leukemias. Blood,92,968-980,1998
4. Kravtsov V, Daniel T, Koury M. Comparative analysis of several methodological approaches to the in vitro studies of cell apoptosis. Am J Pathology,155,1327-1339, 1999.
5. Clarke PG, Clarke S. Historic apoptosis. Nature,378,230,1995
6. Majno G, Joris I. Apoptosis, oncosis, and necrosis. An overview of cell death. Am J Pathol,146,1,3-15,1995
7. Kuprianou N,English HF, Davidson NE, Isaacs JT. Programmed cell death during regression of the MCF-7 human breast cancer following estrogen ablation. Cancer Res,51,162-6,1991
8. Warri AM, Huovinen LR, Laine AM, Martikainen PM, Harkonen PL. Apoptosis in toremifene-induced growth inhibition of human breast cancer cells in vivo and in vitro. J Natl Canc Inst,85,17,1993
9. Krajewski S, Blomqvist C, Franssila K, Krajewska M, et al. Reduced expression of proapoptotic gene Bax is associated with poor response rates to combination chemotherapy and shorter survival in women with metastatic breast adenocarcinoma. Cancer Res,55,4471-78,1995
10. Bergman PJ, Kiefer JA, Price JE, Ley PB, Wynn P, Bucana C, O'Brian CA, McConkey DJ. Acquisition of apoptosis resistance with chemoresistance and metastatic potential in human breast cancer: roles of bcl-2 and bcl-x (L). Proc Annu Meet Am Assoc Cancer Res, 37, A115,1996.
11. Wu J. Apoptosis and angiogenesis: two promising tumor markers in breast cancer. Anticancer Res,16,2233-40,1996
12. Williams GT. Programmed cell death: apoptosis and oncogenesis. Cell,65,1097-98,1991.
13. Marx J. Cell death studies yield cancer clues.Science,259,760-1,1993.
14. Kerr JFR., Winterfold CM, Harmon BV. Apoptosis. Its significance in cancer and cancer therapy. Cancer, 73,8,2013-26,1994.
15. Hannun Y. Apoptosis and the dilemma of cancer chemotherapy. Bllod,89,6,1845-53,1997
16. Wyllie AH, Kerr JFR, Currie AR : Cell death : The significance of apoptosis. Int Rev Cytol 68 : 251, 1980.
17. Gorczyca W, Bruno S, Darzynkiewics RJ, Gong J, Darzynkiewics Z : DNA strand breaks occurring during apoptosis : Their early in situ detection by the terminal deoxynucleotidyl transferase and nick translation assays and prevention by serine protease inhibitors. Int J Oncol 1 : 639, 1992.
18. Kravtsov V, J.P. Greer, Y. Shyr, D.J.Haselton, J.A. Whitlock, S. Goodman, R.S. Stein, M.J. Koury Prediction of survival and responses to chemotherapy in acute myelogeneous leukemia (AML) by the microculture kinetic (MiCK) assay of apoptosis. Blood,92:677a, 1998
19. Kravtsov V, J. Greer, Y. Shyr, J. Whitlock, T., T.McCurley, Goodman, R. Stein, S.Krantz, M. Koury Prediction of survival in acute non-lymphocytic leukemia (AML). Blood,98:214b, 2001
20. Kravtsov V, J. Greer, Y. Shyr, J. Means, J. Whitlock, T., T.McCurley, Goodman, R. Stein, S.Krantz, M. Koury Prospective study evaluating ability of the MiCK assay for apoptosis to predict complete remission and survival in de novo acute myeloid leukemia (manuscript is being submitted for publication).
21. Kravtsov V, V.Priego, J.Reilly, H.Sethi, J.Cooke,W.Smith,M.Koury Chemotherapy of myeloid leukemia directed by a microculture kinetic assay for apoptosis. Blood, 96:3129, 2000.
22. Zang J, Kravtsov V, Amarnath V, Picklo M, Graham D, Montine T. (2000) Enhancement of dopaminergic neurotoxicity by the mercapturate of dopamine: relevance to Parkinson's disease. J Neurochem , 74,970-978.
23. Schultz R, M.Rothenberg, M.Koury, W.Hankins, V.Kravtsov. Sequence dependence using combination of Alimta (pemetrexed disodium, LY231514,MTA), gemcitabine, and oxaliplatin in human colorectal carcinoma cell lines. The 11th NCI-EORTC-AACR Symposium. Clin Cancer Res, 6:1078, 2000.
